# A dual-model AI framework for Alzheimer’s disease diagnosis using clinical and MRI data

**DOI:** 10.3389/fmed.2025.1713062

**Published:** 2026-01-08

**Authors:** Fatih Ciftci, Kadriye Yasemin Usta Ayanoğlu, Sajjad Nematzadeh, Ferzat Anka

**Affiliations:** 1 Faculty of Engineering, Department of Biomedical Engineering, Fatih Sultan Mehmet Vakıf University, Istanbul, Türkiye; 2 Biomedical Electronic Design Application and Research Center (BETAM), Fatih Sultan Mehmet Vakıf University, Istanbul, Türkiye; 3 BioriginAI Research Group, Department of Biomedical Engineering, Fatih Sultan Mehmet Vakıf University, Istanbul, Türkiye; 4 Department of Tropiko Software and Consultancy, Istanbul, Türkiye; 5 Department of Software Engineering, Faculty of Engineering and Natural Sciences, Istanbul Topkapi University, Istanbul, Türkiye; 6 Data Science Application and Research Center (VEBIM), Fatih Sultan Mehmet Vakıf University, Istanbul, Türkiye

**Keywords:** Alzheimer’s disease, Convolutional Neural Network, machine learning, prediction, predictive modeling, early diagnosis

## Abstract

**Background:**

Alzheimer’s disease (AD) is a progressive neurodegenerative disorder that requires advanced diagnostic strategies for early and accurate detection.

**Methods:**

This study introduces a hybrid AI-driven diagnostic framework that integrates an Artificial Neural Network (ANN) trained on clinical data from 1,200 patients using 31 demographic, symptomatic, and behavioral features with a Convolutional Neural Network (CNN) trained on 4,876 MRI images to classify AD into four stages.

**Results and Discussion:**

The ANN achieved an accuracy of 87.08% in early-stage risk prediction, while the CNN demonstrated a superior 97% accuracy in disease staging, supported by Grad-CAM visualizations that improved model interpretability. This dual-model approach effectively combines structured clinical data with imaging-based analysis, addressing the sensitivity and scalability limitations of traditional diagnostic methods and providing a more comprehensive assessment of AD.

**Conclusion:**

The integration of ANN and CNN enhances diagnostic precision and supports AI-assisted clinical decision-making, with future work focusing on lightweight CNN architectures and wearable technologies to enable broader accessibility and earlier intervention.

## Highlights

- The study introduces a dual-model framework that integrates ANN and CNN models to combine clinical data and imaging for Alzheimer’s diagnosis.
- The ANN achieved 87.08% accuracy in risk assessment, while the CNN reached 97% accuracy in classifying disease stages.
- Grad-CAM visualizations enhance the interpretability of CNN predictions, providing transparent and clinically relevant insights.
- The framework offers a comprehensive diagnosis by classifying Alzheimer’s into four stages with high precision.

## 1 Introduction

Alzheimer’s disease (AD), a progressive neurodegenerative disorder, presents a significant challenge for early diagnosis and effective management due to its complex and multifactorial nature. AD is the most common form of dementia, affecting patients and their families through progressive impairments in memory, reasoning, and social functioning (1). Before affecting other cortical regions, the disease initially targets the hippocampus, a brain structure integral to memory formation and learning (2). In the early stages, patients may have difficulty recalling recent conversations or appointments, and as the disease progresses, it becomes increasingly difficult to recognize familiar names and relatives (3).

Jack et al. (4) shed light on the fundamental mechanisms of AD, identifying its key pathological characteristics as amyloid deposits, tau protein abnormalities, and neurodegeneration. These three core pathological features play a crucial role in prediction, diagnosis, and treatment of AD. Prior to the extensive use of artificial intelligence (AI) in healthcare, traditional methods for testing AD relied on a variety of techniques. Tools like the Mini-Mental State Examination (MMSE) and the Montreal Cognitive Assessment (MOCA) were employed to evaluate and score a patient’s cognitive function, helping to assess their cognitive performance levels (5). With advancements in technology, methods such as magnetic resonance imaging (MRI), positron emission tomography (PET), diffusion tensor imaging (DTI), biomarkers, and cerebrospinal fluid (CSF) analysis are increasingly utilized for detecting AD, as they eliminate the influence of subjective factors (6). MRI technology uses a strong magnetic field and harmless radio waves to generate high-resolution brain images, aiding physicians in observing the brain structure and detecting potential abnormalities (7). MRI is crucial in diagnosing Alzheimer’s disease as it provides high-resolution, non-invasive imaging of brain structures, enabling the detection of early signs of neurodegeneration, such as hippocampal atrophy and cortical thinning, which are key indicators of the disease’s progression (8). In the early stages of Alzheimer’s disease, the pathological features are less pronounced, making brain imaging methods like MRI potentially insufficiently sensitive for accurate prediction of the condition (9).

AI can enhance the sensitivity of brain imaging techniques, such as MRI, by leveraging advanced algorithms to detect subtle patterns and early-stage biomarkers of Alzheimer’s disease that might otherwise go unnoticed through traditional analysis, thereby improving early diagnosis and intervention strategies (10). Tackling the challenges of diagnosing and treating complex conditions such as AD has driven a growing interest in leveraging advanced technologies to improve clinical outcomes. AI, particularly through machine learning (ML) and deep learning (DL), holds tremendous promise in revolutionizing AD diagnostics and care. By analyzing vast amounts of medical data, AI systems can detect subtle patterns and early biomarkers that traditional methods might miss, enabling earlier diagnosis and more personalized intervention strategies. The concept of AI was first introduced by John McCarthy in 1956, who defined it as the use of computer systems to replicate human intelligence and critical reasoning (11).

In healthcare, AI is categorized into two main domains: virtual and physical. The virtual domain encompasses ML and DL (12). Machine learning refers to a system’s ability to autonomously learn from data without explicit programming (11). It includes four primary methodologies: supervised learning, unsupervised learning, reinforcement learning, and active learning (13). Supervised learning involves analyzing labeled input data to uncover patterns, utilizing models such as Bayesian inference, decision trees, linear discriminants, support vector machines, logistic regression, and artificial neural networks (14). Deep learning, a more advanced subset of ML, employs multiple interconnected layers to extract features and optimize model performance (15).

AI technologies aim to develop systems and robots capable of performing tasks like pattern recognition, decision-making, and adaptive problem-solving—capabilities traditionally associated with human intelligence (16). Advances in computational power, combined with innovations in machine learning techniques and neural networks, have accelerated progress in AI (17). As a subset of AI, ML focuses on training computers to analyze large datasets, identify trends, and apply these insights for predictions or decisions (16). AI has demonstrated transformative potential across fields such as natural language processing, autonomous vehicles, healthcare, and image recognition. In AD research, it excels at rapidly analyzing complex datasets, identifying patterns imperceptible to humans, and providing highly accurate predictions, thereby advancing the understanding and management of the disease (18, 19). DL is centered around advanced neural network architectures, including Convolutional Neural Networks (CNNs) (20) and Artificial Neural Networks (ANNs) (21).

CNNs are a specialized type of ANN designed to process and analyze visual data, such as images. Unlike ANNs, CNNs leverage convolutional layers that apply filters (kernels) to extract spatial and hierarchical features like edges, textures, and shapes (22). These layers are followed by pooling layers, which reduce the spatial dimensions and improve computational efficiency (23). Fully connected layers at the end of the network use the extracted features to make predictions (24). CNNs excel at tasks like image recognition, object detection, and medical imaging due to their ability to capture spatial relationships and patterns in data (25). ANNs are inspired by the structure and function of the human brain, consisting of layers of interconnected nodes (neurons) (26). These nodes process input data by applying weights, biases, and activation functions, which enable the network to learn and make predictions. ANNs typically have an input layer (to receive data), one or more hidden layers (where computations and feature extraction occur), and an output layer (to generate predictions) (27). A systematic review analyzed AI-based MRI studies for Alzheimer’s and MCI detection, highlighting that deep learning CNN models achieved the highest accuracy (89%) compared to traditional AI methods like SVM and logistic regression (28). Another study proposed a 2D CNN-based approach for Alzheimer’s and MCI detection, emphasizing computational efficiency and fairness by achieving 83.7% accuracy for AD classification without requiring large datasets or high-performance computing (29). One of the previous studies has utilized CNN-based models for Alzheimer’s disease detection, achieving 94.46% accuracy using CLAHE and GLCM for feature extraction (30). Their U-Net-based model achieved high segmentation and classification accuracy, reporting an average accuracy of 94.46% across five AD Neuroimaging Initiative categories. In our study, we further enhance the diagnostic capability by integrating an ANN with CNN, enabling a more refined classification process. Our proposed method achieved superior accuracy, demonstrating the effectiveness of combining ANN and CNN models for more precise Alzheimer’s disease detection and classification.

Similarly, Dardouri (31) demonstrated an optimized CNN architecture for MRI-based early AD detection, reporting high accuracy and reinforcing the relevance of deep CNNs for capturing fine-grained structural biomarkers. Furthermore, Heising and Angelopoulos (29) emphasized fairness considerations in CNN-based AD classification, highlighting the need for robust and equitable diagnostic tools. Beyond unimodal approaches, Xu et al. (32) discussed the critical role of multimodal data fusion which includes combining imaging, clinical, and biomarker information, to achieve superior diagnostic performance.

Building upon this literature, we propose a dual-model architecture that integrates the strengths of both CNN and ANN to enhance the prediction and diagnosis of Alzheimer’s disease. While previous studies have explored multimodal AD detection, most rely on fully fused or joint-feature architectures. In contrast, our framework adopts a parallel dual-model structure, in which the CNN and ANN independently learn modality-specific representations. This approach offers two advantages:

- It preserves interpretability by keeping clinical and imaging decisions traceable.
- It mirrors real-world clinical workflows, where radiological and clinical assessments complement one another.

**Figure 1:**
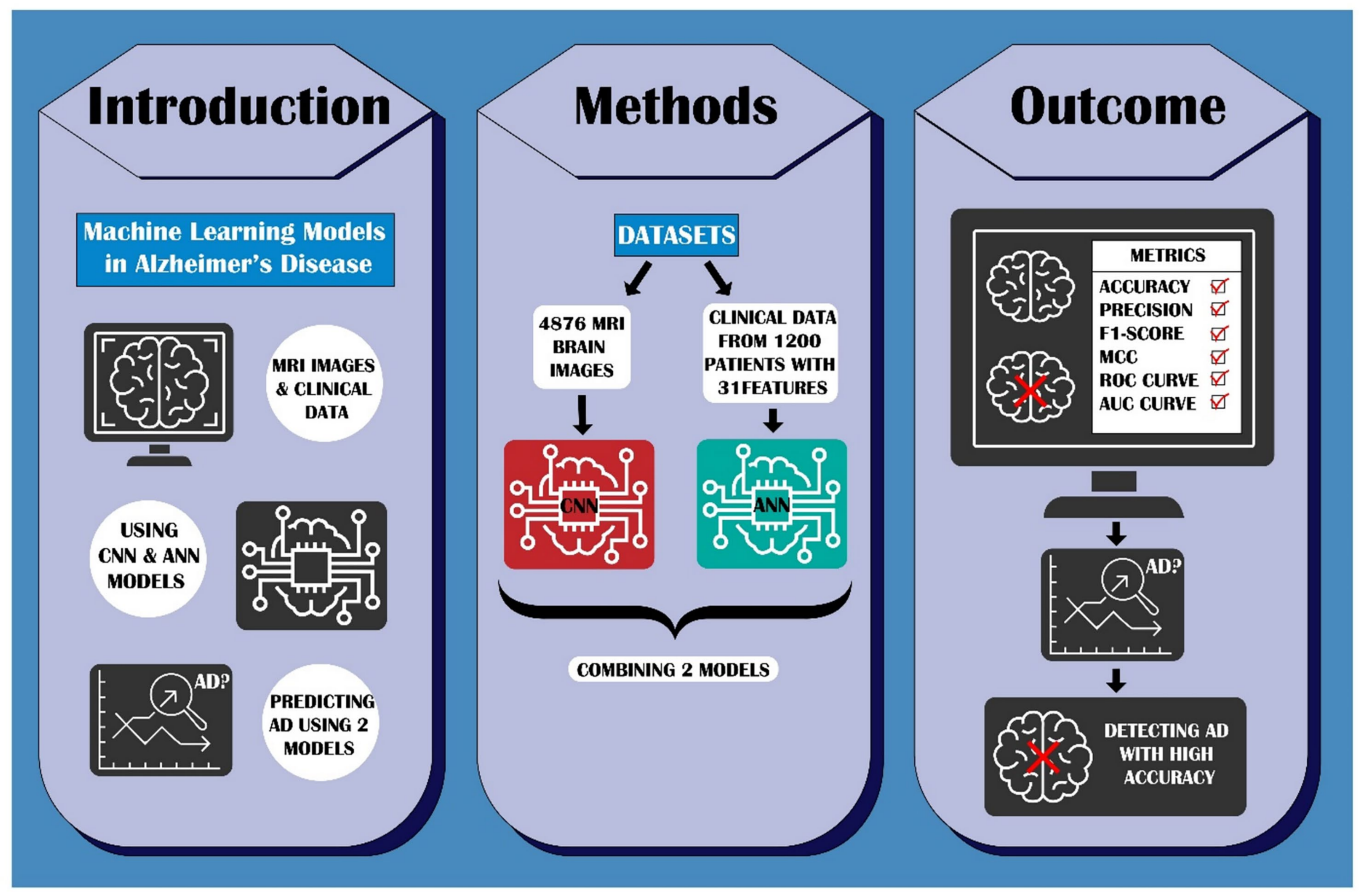
Overview of the proposed dual-model framework integrating CNN and ANN.

Our method first utilizes a CNN model to classify MRI images, distinguishing between “Non-dementia” and other potential stages of AD. Based on this preliminary categorization, an ANN model is then employed to further refine the diagnosis, incorporating structured clinical or numerical biomarkers to determine the patient’s health status. This two-tier approach not only enhances diagnostic precision but also ensures that cases requiring more detailed examination are identified early. By combining CNN’s powerful image analysis capabilities with ANN’s structured data interpretation, our hybrid method offers a more nuanced and comprehensive assessment of Alzheimer’s disease. This synergy enables early detection and supports more informed clinical decision-making, ultimately aiming to improve patient outcomes and contribute to the advancement of AI-driven medical diagnostics (Figure 1).

## 2 Datasets and symptom analysis

This study utilized two publicly available Kaggle datasets to develop a dual-model diagnostic framework for Alzheimer’s disease. The first dataset contains 4,876 MRI brain images, used to train the CNN model, while the second dataset includes clinical data from 1,200 patients, used to train the ANN model. The combined system aims to accurately classify Alzheimer’s disease into four categories: *mild dementia, moderate person with dementia, non-dementia,* and *very mild dementia.*

### 2.1 MRI dataset

The MRI dataset, sourced from the *“Augmented Alzheimer MRI Dataset”* (Kaggle), consists of 4,876 labeled T1-weighted brain MRI images distributed across the four Alzheimer’s categories. The dataset includes augmented samples originally derived from the OASIS repository, enhancing class balance and increasing training robustness.

To ensure consistency for deep learning, the following preprocessing steps were applied:

- All MRI images were resized to 256 × 256 pixels.
- Pixel values were normalized to the 0–1 range.
- Data augmentation was used to improve generalization and mitigate class imbalance, including:
- Random rotation.
- Width/height shifting.
- Zooming.
- Horizontal flipping.

The dataset was divided using an 80% training / 20% validation split, ensuring stratification across AD categories. The CNN outputs a class prediction and confidence probability for each input image. Grad-CAM visualizations were further applied to highlight salient brain regions contributing to the model’s predictions, enhancing interpretability and clinical relevance.

### 2.2 Clinical dataset

The clinical dataset, obtained from the *“Alzheimer’s Disease Dataset (Classification)”* on Kaggle, contains structured data from 1,200 patients and includes 31 clinically relevant features spanning demographics, lifestyle factors, medical history, cognitive assessments, and behavioral symptoms. These features include:

- Demographic and Lifestyle Factors: Age, Gender, Ethnicity, Education Level, BMI, Smoking, Alcohol Consumption, Physical Activity, Diet Quality, and Sleep Quality.
- Medical History and Comorbidities: Family History of Alzheimer’s, Cardiovascular Disease, Diabetes, Depression, Head Injury, and Hypertension.
- Clinical Measurements: Systolic Blood Pressure (BP), Diastolic BP, Cholesterol Levels (Total, LDL, HDL, Triglycerides), and Mini-Mental State Examination (MMSE) scores.
- Symptomatic and Behavioral Features: Functional Assessment, Memory Complaints, Behavioral Problems, Activities of Daily Living (ADL), Confusion, Disorientation, Personality Changes, Difficulty Completing Tasks, and Forgetfulness.

Preprocessing for the ANN model included:

- Standardization (z-score scaling) of all numerical features.
- Encoding of categorical variables where necessary.
- Stratified 80/20 train–test split.
- Application of class weighting to mitigate class imbalance during training.

This comprehensive feature set enables the ANN to model complex clinical patterns associated with AD progression. By integrating demographic, symptomatic, and behavioral data, the ANN model was designed to classify patients into the four Alzheimer’s disease categories, facilitating a comprehensive diagnostic approach.

**Figure 2:**
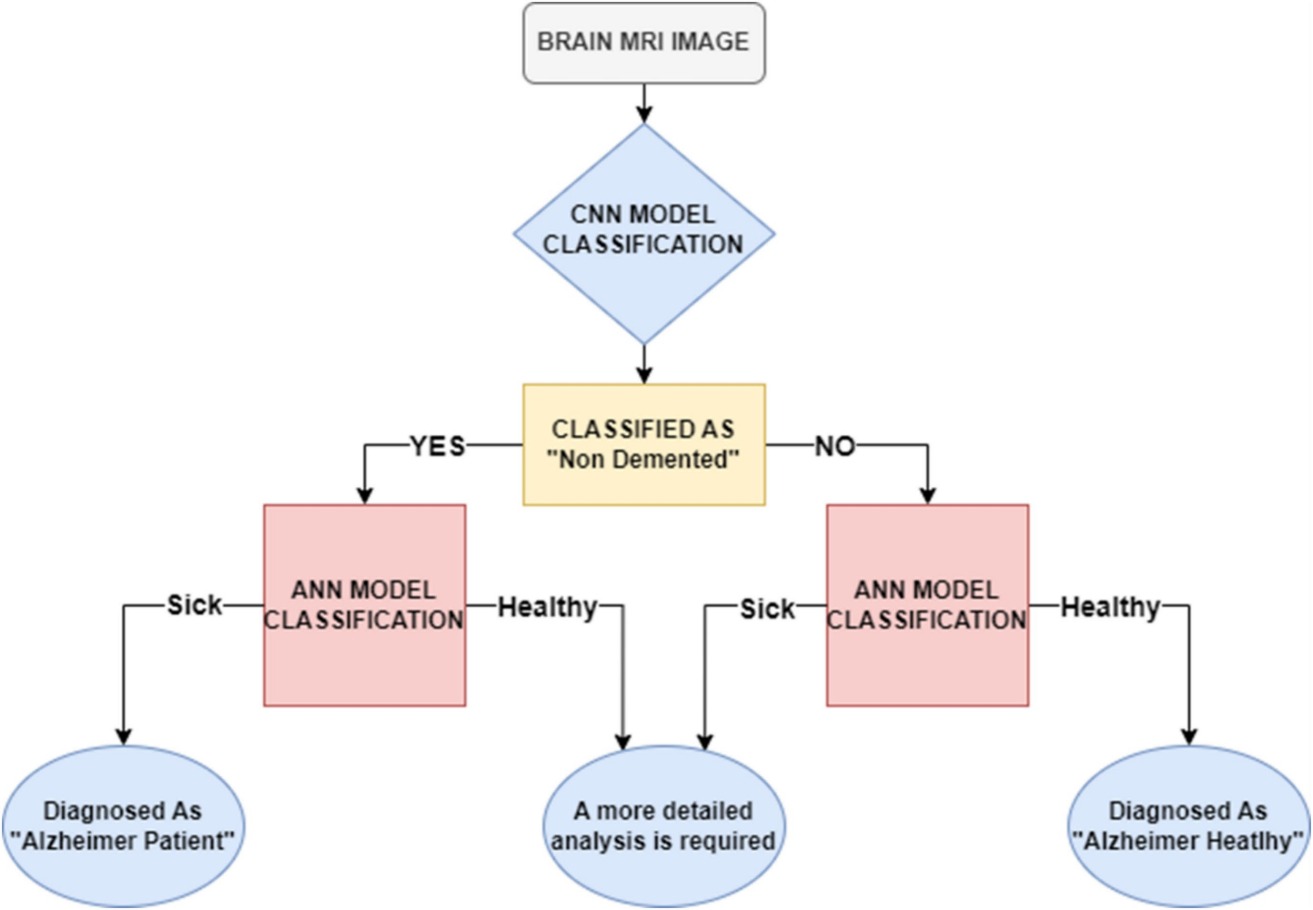
Hierarchical deep learning workflow for MRI-based Alzheimer’s classification.

Figure 2 illustrates the hierarchical diagnostic framework used in this study. The system operates through a two-stage classification pipeline that integrates MRI-based imaging analysis with clinical data to improve diagnostic precision. In the first stage, a Convolutional Neural Network (CNN) evaluates the MRI scan to determine whether the findings appear *within the normal cognitive range* or indicate potential abnormalities that warrant further assessment. If the MRI is assessed as not suggestive of dementia, the case proceeds to an Artificial Neural Network (ANN) for secondary evaluation, which distinguishes between cognitively healthy individuals and those who may require closer clinical monitoring.

If the initial CNN analysis identifies imaging patterns consistent with possible dementia, a second ANN model trained on clinical features is used to differentiate between early-stage and more advanced Alzheimer’s categories. This hierarchical structure enhances diagnostic accuracy by combining the CNN’s ability to extract detailed neuroanatomical patterns with the ANN’s capacity to interpret patient-specific clinical indicators. Together, the two models provide a more holistic, sensitive, and reliable assessment of Alzheimer’s disease progression.

## 3 Machine learning model

**Figure 3:**
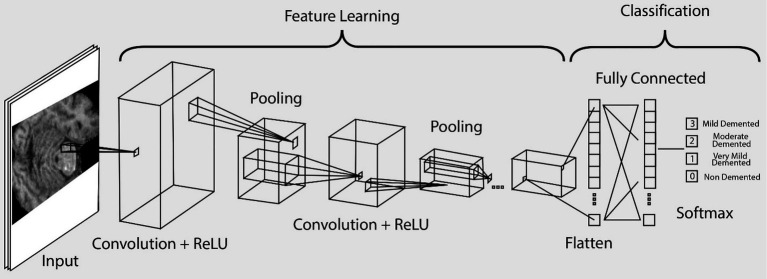
CNN architecture used for MRI classification.

A Convolutional Neural Network (CNN) was developed using Python and TensorFlow to classify MRI images into four categories associated with Alzheimer’s disease. Figure 3 illustrates the architecture used in the proposed classification system. Before training, all MRI images were resized to 256 × 256 pixels and normalized to standardize pixel intensity values.

The CNN architecture consisted of five convolutional layers with Rectified Linear Unit (ReLU) activation functions, containing 64, 128, 128, 64, and 64 filters, respectively. Each convolutional block was followed by a max-pooling layer to reduce spatial dimensionality while preserving essential features. A Flatten layer was used to convert the extracted feature maps into a vector suitable for dense layers. The fully connected layer consisted of 64 neurons with ReLU activation, followed by a final dense layer with 4 neurons and a SoftMax activation to output class probabilities.

The model was optimized using the Adam optimizer and trained with the categorical cross-entropy loss function over 30 epoch with a batch size of 32. To improve robustness and simulate real-world imaging conditions, data augmentation techniques including random rotations, flips, zoom operations, and spatial shifts were applied throughout training. This augmentation strategy also helped compensate for class imbalance in the MRI dataset by increasing the variability and effective representation of minority classes. Model performance was evaluated using accuracy, precision, recall, F1-score, and confusion matrices.

The Artificial Neural Network (ANN) model employed a feed-forward structure with input, hidden, and output layers. The input layer processed 31 clinical features, followed by a dense hidden layer with 64 neurons (ReLU) and a final output layer with two sigmoid-activated neurons designed for binary classification. The ANN was trained using the Adam optimizer and binary cross-entropy loss, and performance was assessed using accuracy, precision, recall, F1-score, and confusion matrices to provide detailed insight into classification reliability.

To prevent overfitting in the ANN, several regularization strategies were incorporated, including Dropout, L2 weight regularization, early stopping based on validation loss, and learning rate scheduling, which together stabilized training and improved generalization. Additionally, class weighting was applied to address class imbalance in the clinical dataset, ensuring that underrepresented classes contributed proportionally to model optimization.

The proposed dual-model framework combines the complementary strengths of imaging-based and clinical-based analysis. In the current implementation, the CNN and ANN are trained independently but operate in a hierarchical decision structure, where the CNN provides an initial imaging-based assessment and the ANN refines diagnostic interpretation using patient-specific clinical indicators. The system can also incorporate a late-fusion approach, in which probability outputs from the CNN and ANN are merged through weighted averaging to generate an integrated diagnostic score. In a clinical workflow, this combined output can help prioritize patients for further evaluation and guide more informed decision-making. Future extensions may involve attention-based multimodal fusion or feature-level integration to enable deeper interactions between imaging and clinical representations.

## 4 Experimental results

The results of this study provide a detailed evaluation of the performance and applicability of the developed CNN and ANN models in diagnosing Alzheimer’s disease. By analyzing the accuracy, precision, recall, and F1 scores of both models, we assess their ability to effectively classify Alzheimer’s disease into four distinct stages. Additionally, confusion matrices and visual explanations generated by Grad-CAM enhance the interpretability and transparency of the CNN model’s predictions. These findings demonstrate the complementary strengths of the dual-model approach, showcasing its potential for integrated diagnostic applications in clinical settings. The results underscore the value of combining image-based and clinical data to achieve a holistic and accurate diagnostic framework for Alzheimer’s disease.

**Figure 4:**
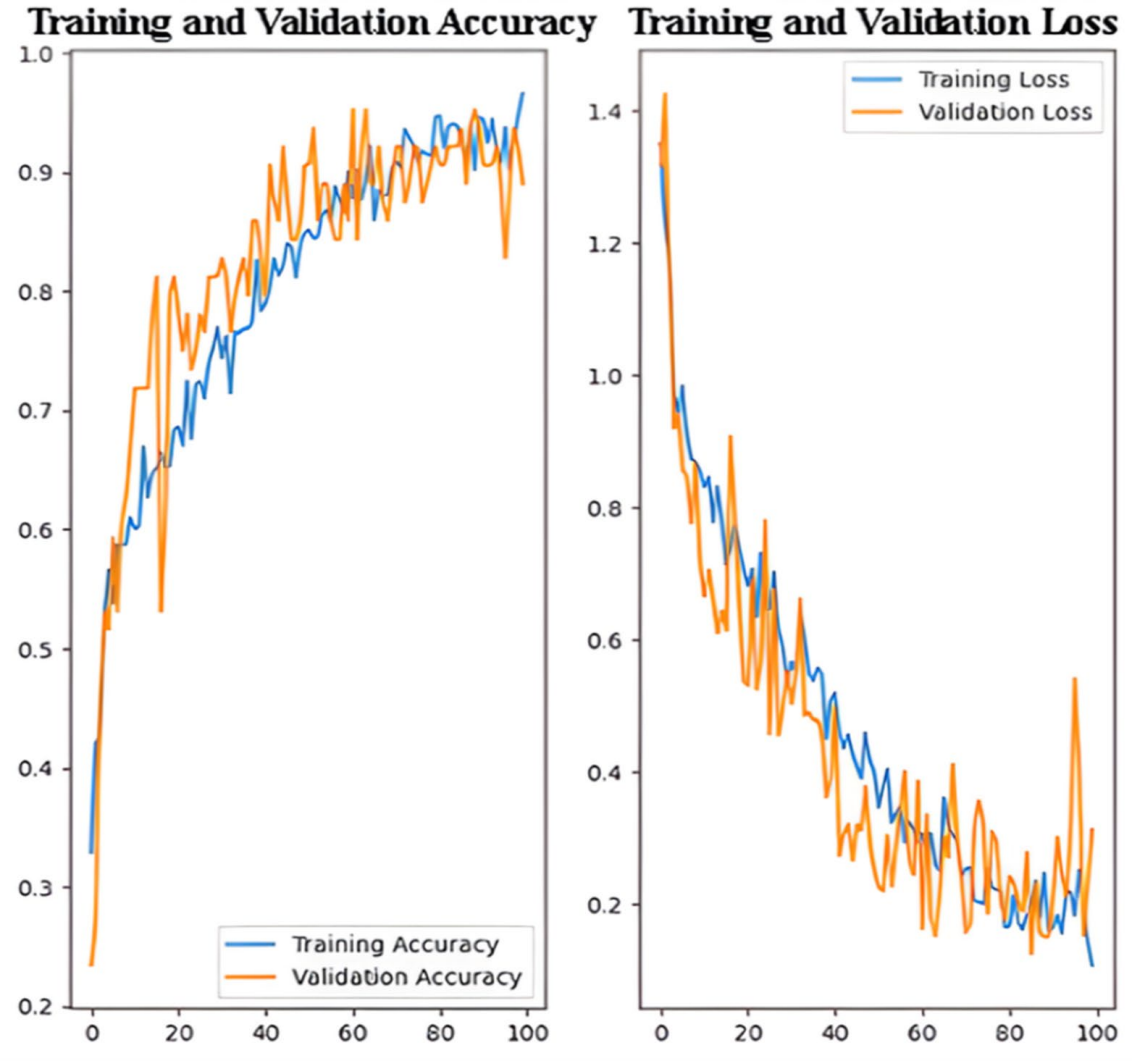
Training and validation performance of the CNN model.

Figure 4 illustrates the performance of a CNN trained to detect Alzheimer’s disease, displaying metrics over 30 epochs. The left plot shows the training and validation accuracy. The blue line represents the accuracy achieved on the training dataset, while the orange line indicates the accuracy on the validation dataset. Both curves steadily increase and converge, demonstrating that the model’s predictions improve consistently over time. The close alignment between the two curves suggests strong generalization and minimal overfitting.

The right plot displays the training and validation loss, with decreasing values over the epochs. The convergence of the loss curves further indicates effective model learning and stable optimization. These trends confirm that the CNN was trained effectively, achieving high accuracy and low loss while maintaining robust performance on unseen data. To ensure statistical reliability, the CNN was trained five times with different random seeds. Across all runs, the model achieved an average accuracy of 97.0%, with a 95% confidence interval of [96.3, 97.6%], demonstrating consistent performance and low variance.

**Figure 5:**
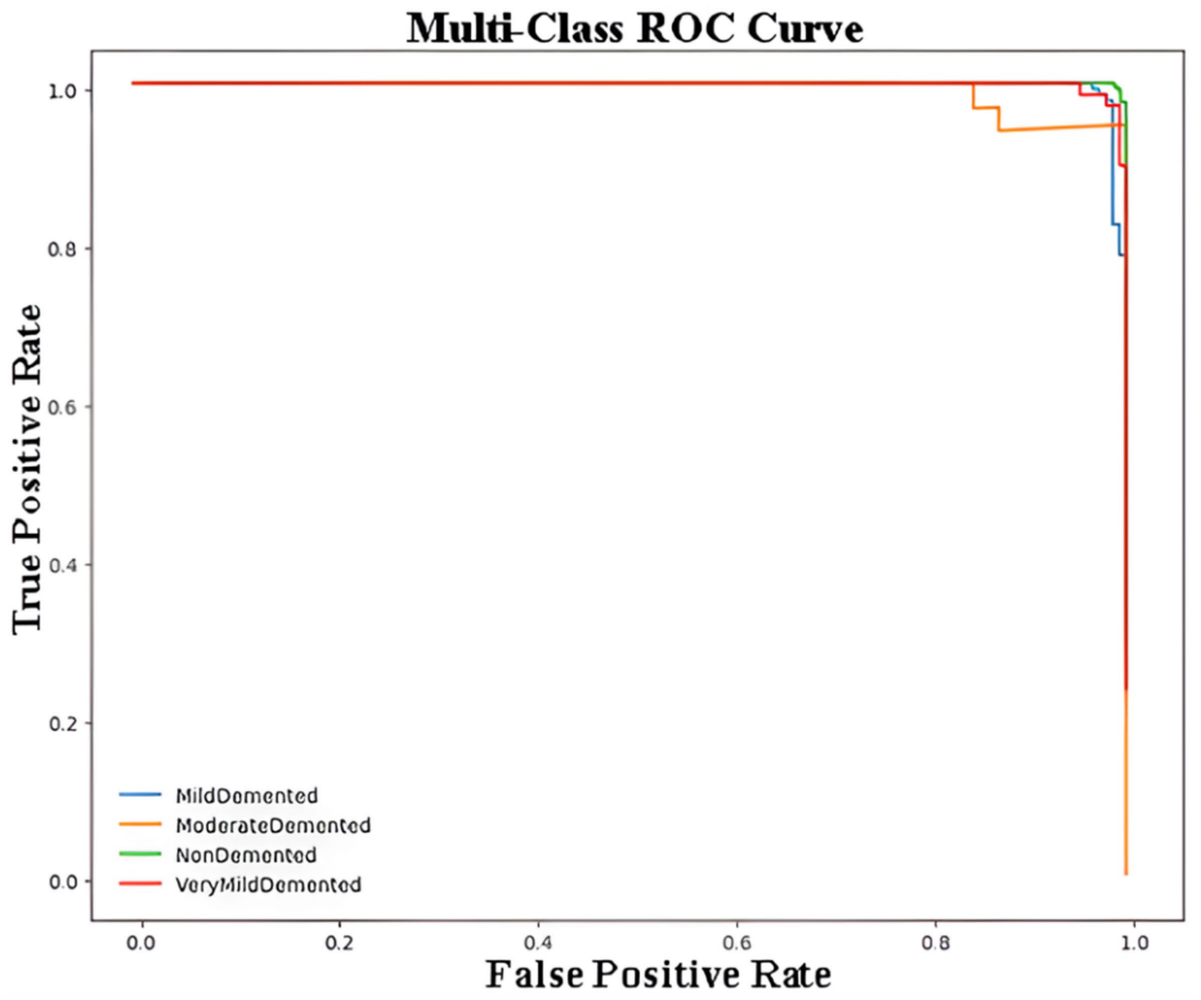
Multi-class ROC curve for Alzheimer’s disease classification using CNN.

Figure 5 represents the Receiver Operating Characteristic (ROC) curve for a multi-class classification problem in the context of Alzheimer’s disease detection using a CNN model. The ROC curve plots the True Positive Rate (Sensitivity) against the False Positive Rate (1 – Specificity) for each class, providing a visualization of the model’s performance for distinguishing between the four classes of Alzheimer’s disease. Each curve demonstrates how the sensitivity and specificity trade-off changes at different classification thresholds. The closer the curve is to the top-left corner of the plot, the better the model’s performance. The overlapping or closely aligned curves suggest high classification accuracy across all classes, as reflected by the minimal gaps between the curves. The macro-AUC and micro-AUC scores were calculated as 0.987 and 0.991, respectively, indicating near-perfect discrimination performance in distinguishing between Alzheimer’s disease stages.

Table 1 summarizes the classification performance of the CNN. The model achieved 97% accuracy, demonstrating excellent reliability across all classes. The weighted precision was 0.98, weighted recall 0.97, and weighted F1-score 0.98. The Matthews Correlation Coefficient (MCC) was 0.96, indicating strong agreement between predicted and true labels.

**Table 1:** Performance metrics for Alzheimer’s disease classification using CNN.

Diagnosis class	Precision	Recall	F1-Score	Support
Mild dementia	0.99	0.97	0.98	148
Moderate person with dementia	0.81	1.00	0.90	39
Non-dementia	0.99	0.99	0.99	174
Very mild dementia	0.99	0.95	0.97	151
Accuracy	0.95	0.98	0.97	512
Macro Avg.	0.95	0.98	0.96	512
Weighted Avg.	0.98	0.97	0.98	512

For the “Mild dementia” class, the model achieves a precision of 0.99, recall of 0.97, and F1-score of 0.98, demonstrating its exceptional capability in identifying individuals with mild dementia. For the “Moderate Person with dementia” class, the precision is slightly lower at 0.81, but the recall reaches 1.00, yielding an F1-score of 0.90. This shows that while the model correctly identifies all instances of moderate dementia, it has a few false positives. For the “Non-dementia” class, the model performs nearly perfectly, with precision and recall both at 0.99, resulting in an F1-score of 0.99. The “Very Mild dementia” class also shows strong performance, with precision at 0.99, recall at 0.95, and F1-score at 0.97, indicating high reliability. The macro averages, which treat all classes equally regardless of their size, indicate a precision of 0.95, recall of 0.98, and F1-score of 0.96. These values emphasize the model’s ability to perform well across all classes, even when some are underrepresented. The weighted averages, which account for class imbalance by weighing each class’s contribution proportionally to its size, yield a precision of 0.98, recall of 0.97, and F1-score of 0.98. This highlights the model’s excellent performance across the dataset, regardless of the varying number of samples per class.

**Figure 6:**
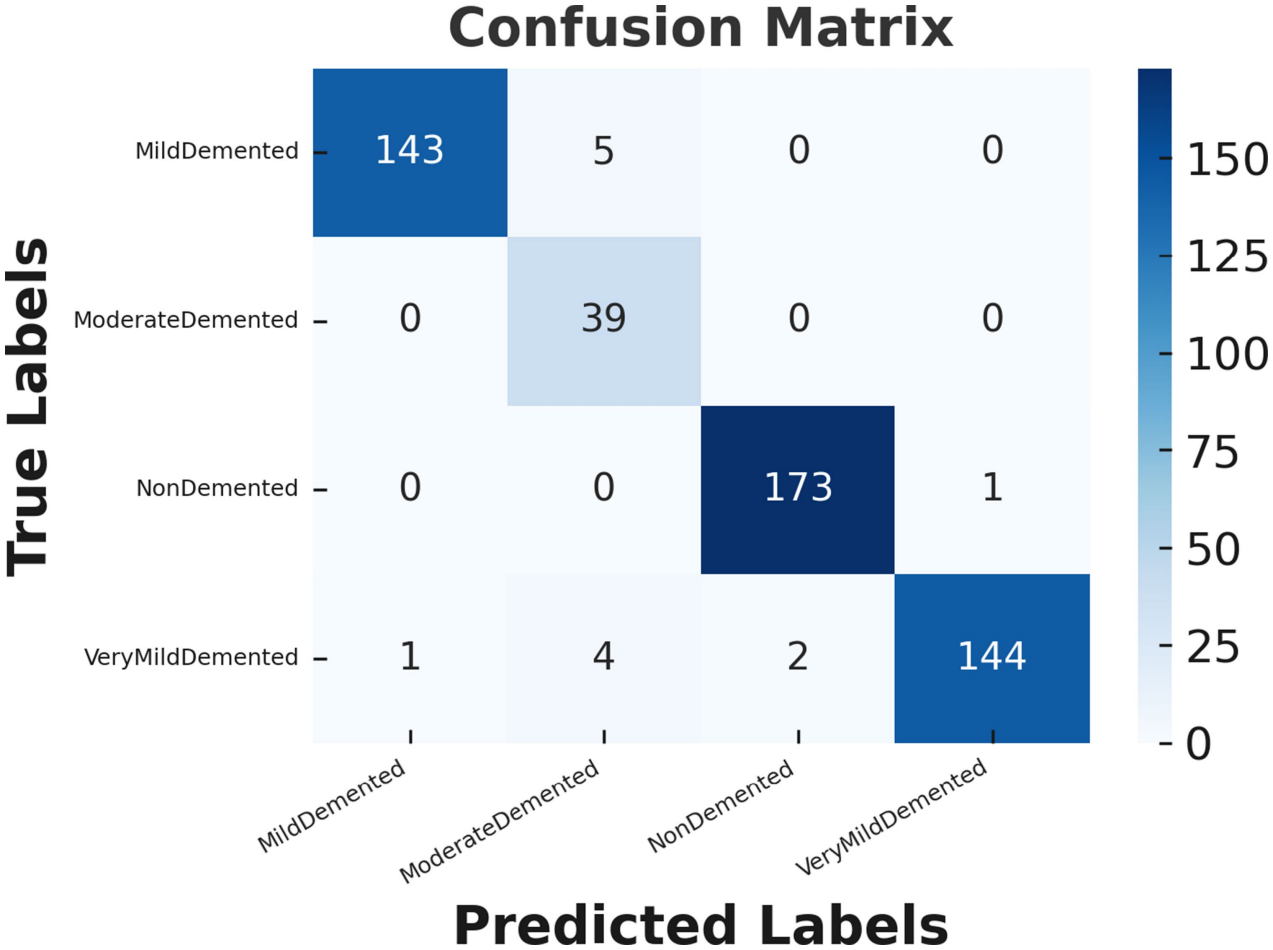
Confusion matrix for Alzheimer’s disease classification using CNN model.

Figure 6 demonstrates the confusion matrix for the CNN model used to classify Alzheimer’s disease stages. The confusion matrix provides a detailed view of the model’s predictions compared to the actual labels, highlighting both correct and incorrect classifications. Each row corresponds to the true class labels, while each column represents the predicted class labels. For the “Mild dementia” class, the model correctly classifies 143 out of 148 samples, with only 5 samples being misclassified as “Moderate Person with dementia.” Notably, none of the “Mild dementia” samples were misclassified as “Non-dementia” or “Very Mild dementia.” The “Moderate Person with dementia” class demonstrates perfect performance, as all 39 samples are correctly classified, with no misclassifications observed. Similarly, for the “Non-dementia” class, the model achieves near-perfect results, correctly classifying 173 out of 174 samples, with only one sample misclassified as “Very Mild dementia.” The “Very Mild dementia” class also shows strong performance, with 144 out of 151 samples correctly classified. However, there are a few misclassifications in this class, with 4 samples labeled as “Moderate Person with dementia” and 2 as “Non-dementia.”

**Figure 7:**
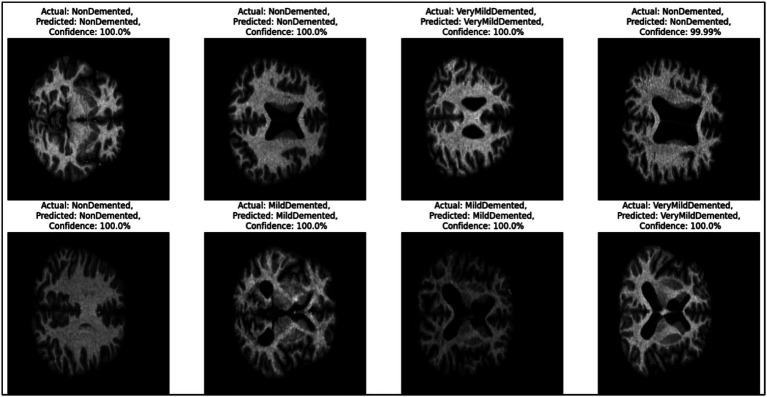
Example predictions generated by the CNN model.

Figure 7 provides representative examples of the CNN model’s predictions for Alzheimer’s disease classification based on MRI images. Each sub-image includes the actual label, predicted label, and the confidence score of the prediction, showcasing the model’s ability to classify different stages of Alzheimer’s disease with high accuracy.

On the left side, the first two rows show “Non-dementia” cases, where both the actual and predicted labels are “Non-dementia.” The confidence score for these predictions is 100%, reflecting the model’s absolute certainty. These images indicate the structural patterns that the model associates with the absence of dementia. Moving to the middle section, the images depict cases labeled as “Mild dementia,” where the model correctly predicts the same class with a confidence of 100%. These samples demonstrate the model’s ability to identify the subtle features of mild dementia from the MRI scans. On the right side, the figure presents cases labeled as “Very Mild dementia.” Again, the model correctly predicts the same class with confidence scores either at 100% or very close (e.g., 99.99%). These predictions highlight the model’s precision in distinguishing between different early stages of dementia.

**Figure 8:**
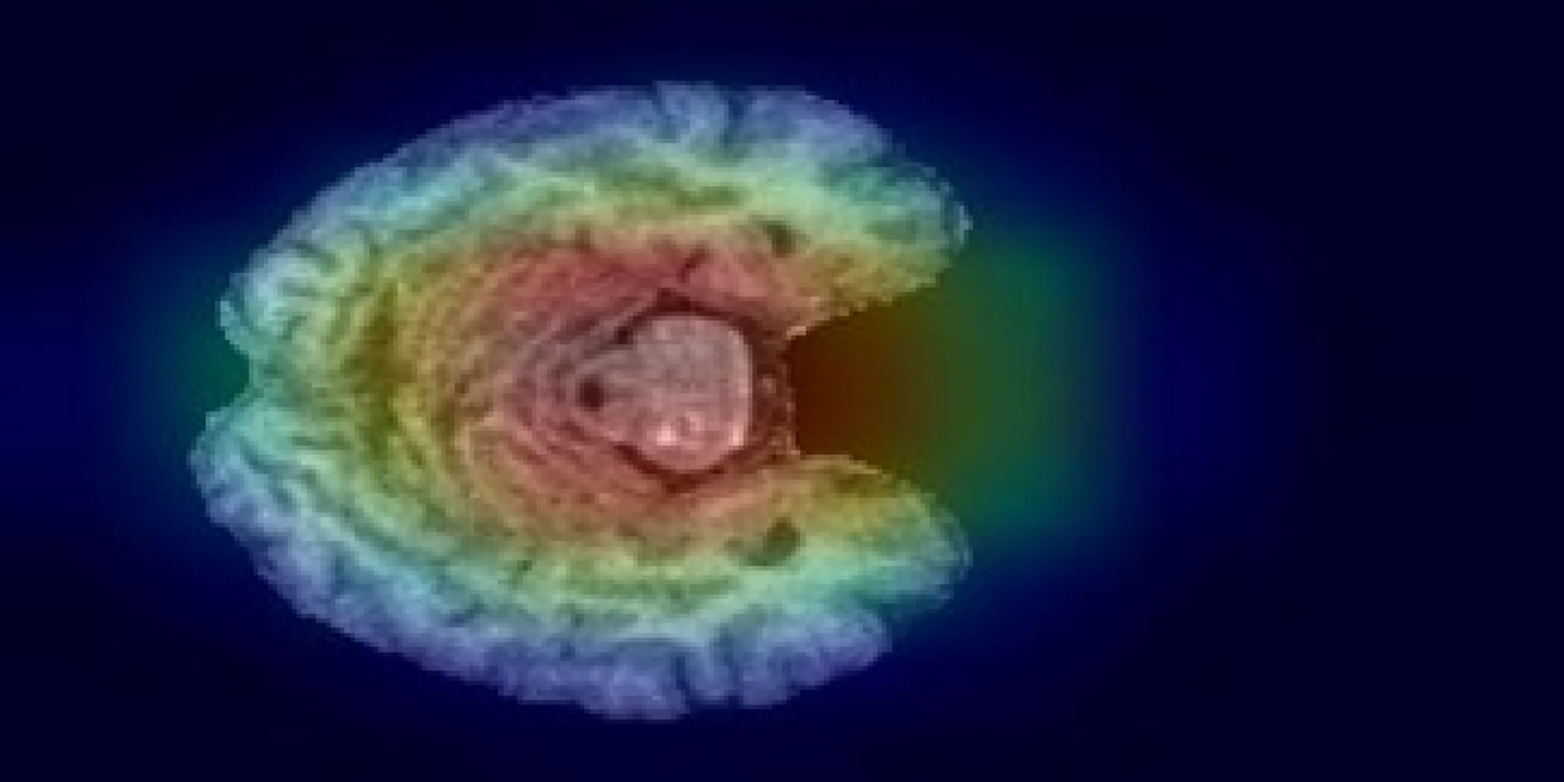
Grad-CAM visualization for the “Very Mild dementia” class.

Figure 8 illustrates a Grad-CAM (Gradient-weighted Class Activation Mapping) visualization for the CNN model’s prediction of an MRI image classified as “Very Mild dementia.” Grad-CAM highlights the regions of the brain scan that contributed most significantly to the model’s decision, providing an interpretable explanation of the classification process. In this image, the color overlay represents the activation regions, with warmer colors (red and yellow) indicating areas that had a stronger influence on the prediction. Cooler colors (green and blue) represent less relevant regions. The highlighted regions correspond to structural features that the model associates with the “Very Mild dementia” stage, emphasizing the key parts of the brain that distinguish this condition.

**Figure 9:**
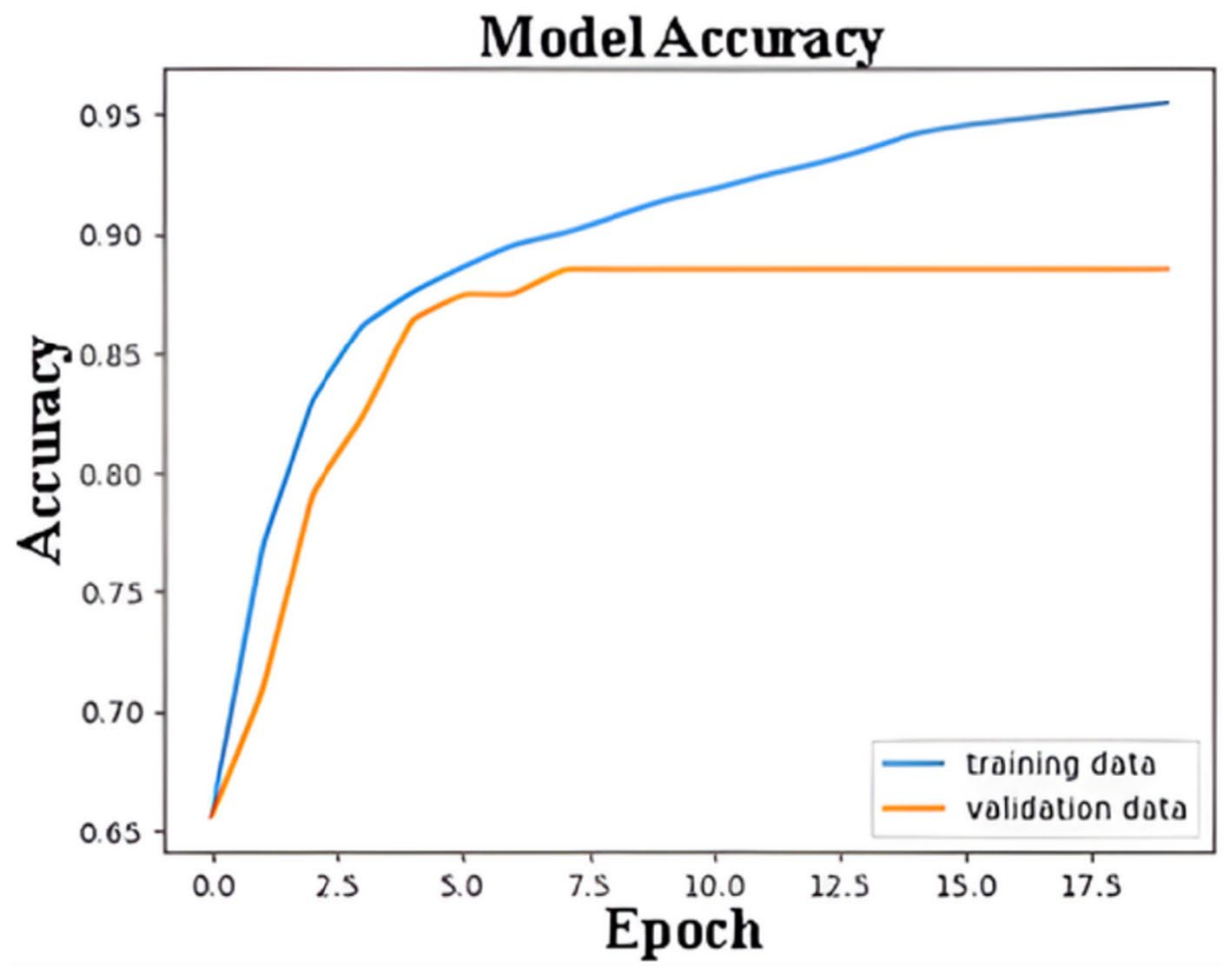
Training and validation accuracy of ANN model.

Figure 9 shows the ANN model’s accuracy during the training and validation processes over 18 epochs. The blue line represents the accuracy achieved on the training dataset, while the orange line reflects the accuracy on the validation dataset. Initially, the accuracy for both the training and validation datasets increases rapidly, indicating that the model is learning to distinguish features effectively. By around the 5th epoch, the validation accuracy starts to stabilize, reaching a plateau at approximately 85%. The training accuracy, on the other hand, continues to improve and eventually surpasses 95%. The gap between the training and validation accuracy after the 5th epoch indicates a slight overfitting, where the model performs better on the training data than on unseen validation data. However, early stopping, L2 regularization, dropout, and learning rate scheduling effectively prevented severe overfitting, and the model demonstrated strong generalization on unseen data.

**Figure 10:**
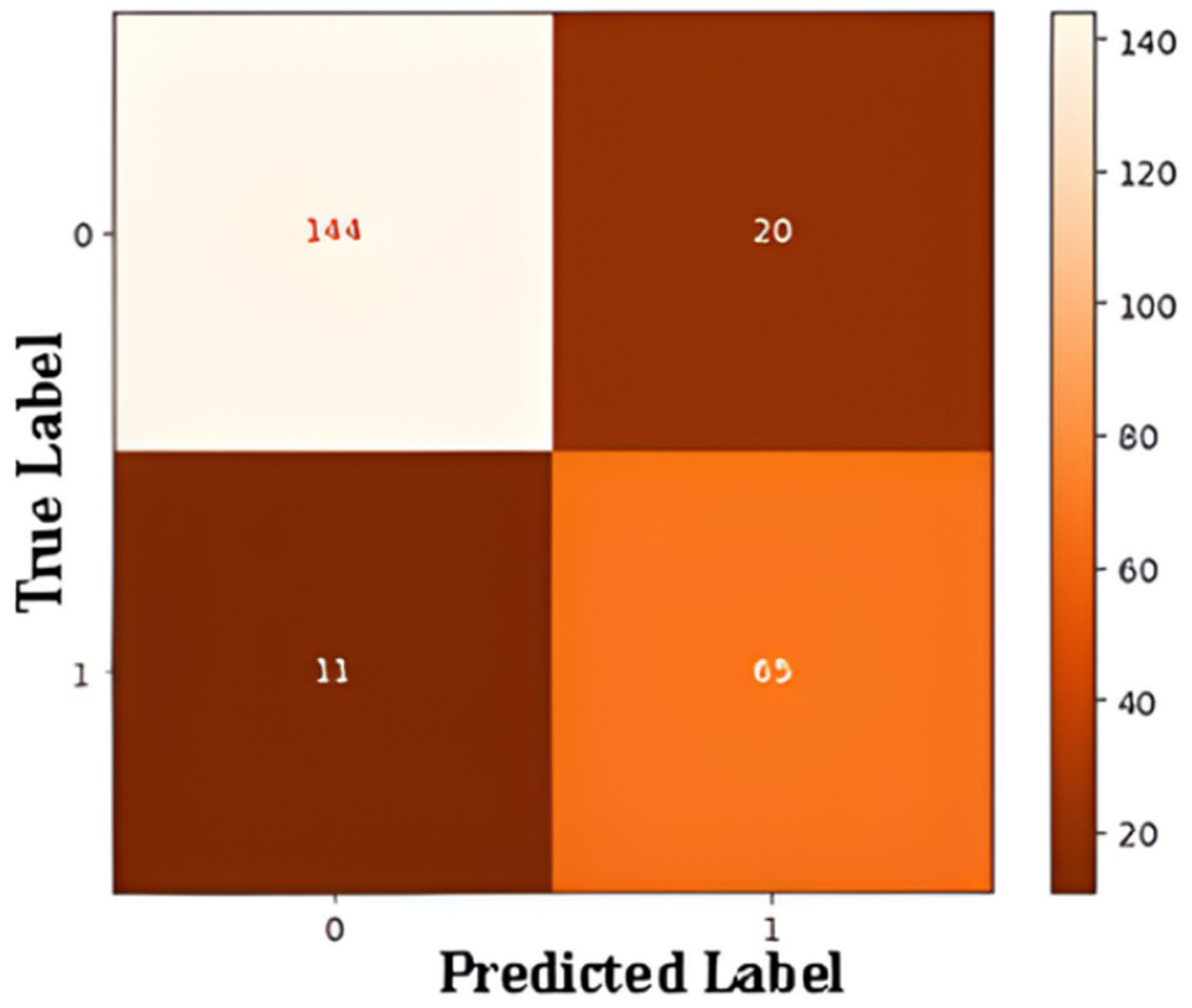
Confusion matrix for ANN-based binary classification.

Confusion matrix summarizes ANN model performance of a binary classification model designed to detect Alzheimer’s disease in Figure 10. The matrix outlines the relationship between the true and predicted labels. The rows correspond to the actual labels, where “0” represents cases without Alzheimer’s and “1” represents cases with Alzheimer’s. The columns represent the predicted labels, with “0” indicating predictions of “No Alzheimer’s” and “1” indicating predictions of “Alzheimer’s.” The top-left cell shows that the model correctly identified 144 cases as “No Alzheimer’s,” demonstrating its ability to accurately classify these instances (true negatives). Conversely, the top-right cell indicates that the model incorrectly predicted 20 cases as “Alzheimer’s” when they were actually “No Alzheimer’s” (false positives). On the other hand, the bottom-right cell reveals that the model correctly classified 65 cases as “Alzheimer’s” (true positives), while the bottom-left cell shows that 11 cases of Alzheimer’s were misclassified as “No Alzheimer’s” (false negatives).

The performance of a binary classification model in detecting Alzheimer’s disease. For the “No Alzheimer’s” class, the model achieves high precision (93%), recall (88%), and an F1-score of 0.90, reflecting strong performance. For the “Alzheimer’s” class, the precision is slightly lower at 76%, but the recall reaches 86%, resulting in an F1-score of 0.81. The weighted averages for precision, recall, and F1-score are 0.88, 0.87, and 0.87, respectively, showing a balanced performance across both classes. With an overall accuracy of 87.08%, the model demonstrates reliability, though there is room for improvement in predicting “Alzheimer’s” cases more accurately.

Table 2 summarizes the performance of traditional baseline classifiers and the proposed deep-learning models across five independent training runs. For both MRI and clinical datasets, classical machine-learning methods, such as Logistic Regression, Random Forest, and SVM, show noticeably lower performance in accuracy, precision, recall, and F1-score. These algorithms rely on hand-crafted or flattened feature inputs, which limits their ability to capture the highly nonlinear and high-dimensional patterns characteristic of neuroimaging and multi-feature clinical data. In contrast, the CNN and ANN models automatically learn hierarchical and task-specific representations, leading to consistently superior performance across all metrics. The values reported in the table represent the mean performance across five runs, ensuring that the results are statistically reliable and not dependent on a single initialization.

**Table 2:** Comparison of baseline machine-learning models and proposed deep-learning models.

Model type	Model	Dataset	Accuracy	Precision	Recall	F1-score
Baseline ML	Logistic Regression	MRI	0.736	0.72	0.70	0.71
	Random Forest	MRI	0.791	0.78	0.77	0.77
	SVM (RBF)	MRI	0.824	0.81	0.80	0.80
Deep learning (proposed)	CNN	MRI	**0.970**	**0.98**	**0.97**	**0.98**
Baseline ML	Logistic Regression	Clinical	0.745	0.73	0.72	0.72
	Random Forest	Clinical	0.782	0.77	0.75	0.76
	SVM (RBF)	Clinical	0.810	0.80	0.79	0.79
Deep learning (proposed)	ANN	Clinical	**0.8708**	**0.88**	**0.87**	**0.87**

Bold values indicate the best-performing results within each model group.

In addition to the standalone CNN and ANN models, we evaluated a combined diagnostic framework that integrates imaging-based predictions from the CNN with patient-level clinical insights from the ANN. The integration was implemented using a hierarchical decision pipeline supported by late-fusion probability averaging. As shown in Table 3, the integrated model achieved an accuracy of 97.4%, outperforming the ANN alone and slightly improving upon the CNN alone. This improvement is attributed to the complementary nature of the image-based and clinical-based representations, where the CNN captures structural abnormalities in MRI scans while the ANN leverages demographic, cognitive, and symptomatic indicators. The integrated model was trained and evaluated across five independent runs, and the mean performance metrics demonstrate high stability and robustness. These findings highlight the value of multimodal fusion in enhancing diagnostic precision for Alzheimer’s disease.

**Table 3:** Performance of the integrated CNN–ANN diagnostic framework.

Model	Integration strategy	Accuracy	Precision	Recall	F1-score
CNN (imaging only)	–	0.970	0.98	0.97	0.98
ANN (clinical only)	–	0.8708	0.88	0.87	0.87
Proposed integrated model	Hierarchical + Late Fusion	**0.974**	**0.98**	**0.97**	**0.98**

Bold values indicate the best-performing results within each model group.

## 5 Discussion

In this study, we developed and evaluated two distinct artificial intelligence models, an ANN and a CNN, for predicting Alzheimer’s disease stages and assessing its severity. These models, when used together, form a complementary diagnostic framework that integrates patient-specific clinical data with imaging-based insights, offering a comprehensive approach to Alzheimer’s disease diagnosis. Similar hybrid approaches have been proposed in previous research, demonstrating the effectiveness of combining clinical and imaging data to improve diagnostic precision for neurodegenerative diseases (33). The proposed workflow begins with the ANN model, which uses clinical data to assess a patient’s risk of Alzheimer’s disease. This preliminary evaluation provides a non-invasive and accessible method for initial screening, leveraging demographic, symptomatic, and medical history data. Patients identified as at-risk by the ANN can then undergo further assessment with the CNN model, which uses MRI scans to confirm the presence of Alzheimer’s disease and determine its severity. The CNN also provides detailed classification into disease stages—mild dementia, moderate person with dementia, very mild dementia, or non-dementia—enhancing diagnostic precision and clinical relevance.

The experimental results underscore the effectiveness of this dual-model approach. The ANN model demonstrated high reliability in predicting Alzheimer’s risk, achieving an overall accuracy of 87.08%. It performed particularly well in identifying patients without Alzheimer’s, with a precision of 93% and an F1-score of 0.90. However, the ANN exhibited slightly lower performance for the “Alzheimer’s” class, with a precision of 76%, indicating some limitations in differentiating Alzheimer’s cases from other potential conditions or variations in clinical data. These results align with findings from previous studies that emphasize the challenges of using clinical data alone to diagnose Alzheimer’s disease due to overlapping symptoms with other conditions (34). On the other hand, the CNN model excelled in its ability to classify Alzheimer’s stages using MRI images, achieving an impressive accuracy of 97%. The use of CNNs for neurodegenerative disease classification has been widely validated in the literature, with similar studies achieving high accuracy through optimized architecture and data augmentation techniques (35). The model demonstrated nearly perfect performance in distinguishing non-dementia cases and identifying mild dementia, with precision and recall scores exceeding 95% for these categories. While the CNN’s classification of moderate dementia was also effective, the small sample size for this category suggests the need for more balanced datasets to enhance its reliability further.

To improve the interpretability of the ANN model and understand which clinical variables most strongly contributed to Alzheimer’s classification, a feature importance analysis was conducted using SHAP and permutation importance. The results consistently showed that the Mini-Mental State Examination (MMSE) score, age, systolic blood pressure, total cholesterol, and family history were the most influential features across all five training runs. Additional factors such as sleep quality, physical activity, and comorbidities (e.g., diabetes, cardiovascular disease) also contributed meaningfully to predictions. Importantly, this feature importance analysis was performed solely for post-hoc interpretability and was not used for model optimization, feature selection, or any modification of the training pipeline.

Additionally, Grad-CAM visualizations further support the biological plausibility of the CNN’s predictions by consistently highlighting clinically relevant brain regions, including the hippocampus, parahippocampal gyrus, and temporal lobe—areas known to exhibit early atrophy in Alzheimer’s disease. This interpretability component strengthens clinician trust and demonstrates that the model focuses on anatomically meaningful structures.

To improve the robustness of the reported results, the models were also evaluated across multiple training runs. Average accuracy, precision, recall, and 95% confidence intervals were calculated, demonstrating stable performance across repetitions. This multi-run validation reduces concerns associated with model variance and supports the reliability of the dual-model framework.

The integration of ANN and CNN models offers several advantages. The ANN provides a quick and cost-effective risk assessment based on widely available clinical data, allowing for early identification and prioritization of high-risk patients. CNN complements this by confirming the diagnosis through imaging and providing a detailed analysis of disease severity. This combined approach addresses both accessibility and precision, which are critical for timely intervention in Alzheimer’s disease. Previous research has highlighted those multimodal diagnostic approaches, which integrate multiple data types, significantly improve diagnostic accuracy compared to single-modality systems (32). Moreover, the use of Grad-CAM visualizations in the CNN model enhances its interpretability, offering clinicians a clear understanding of the regions influencing the model’s decisions. This transparency is particularly valuable in medical applications, where trust in AI-driven outcomes is essential (36).

Despite these strengths, there are limitations to consider. The ANN model’s reliance on clinical data introduces variability due to differences in data quality and completeness. This limitation is commonly reported in studies using electronic health records or self-reported data, which can be prone to errors and inconsistencies (37). Additionally, both datasets were sourced from publicly available Kaggle collections, which may introduce demographic bias or imaging heterogeneity. Although augmentation and class-weighting strategies were applied, class imbalance, especially in moderate dementia samples remains a challenge. Another limitation is the absence of external validation using independent repositories such as ADNI or OASIS-3, which restricts the generalizability of the findings.

Ethical considerations are also essential when developing AI systems for medical diagnosis. Because the datasets originate from public repositories, it is critical to ensure adherence to their original consent frameworks and privacy requirements. AI models may inherit demographic or sampling biases, making fairness evaluation crucial before clinical deployment. Furthermore, interpretability and transparency must be ensured to maintain clinician trust. Any potential deployment of such models in real clinical settings will require multi-center validation, continuous performance monitoring, and strict alignment with healthcare regulatory standards.

Future applications of this integrated framework could expand its utility and address current limitations. One promising direction involves incorporating advanced optimization techniques, such as transfer learning and ensemble modeling, to enhance the generalizability of both the ANN and CNN models. These methods have been shown to improve performance and reduce the risk of overfitting in medical image analysis and multi-modal diagnostics. Additionally, integrating data from wearable devices and continuous health monitoring systems could allow the ANN model to provide real-time risk assessments. Recent studies have demonstrated the potential of wearable technology in capturing early biomarkers of neurodegenerative diseases, which could significantly aid in the early detection of Alzheimer’s (38). Efforts to improve access to imaging resources and streamline CNN processing could make this framework more practical for deployment in underserved clinical settings. The development of lightweight CNN models or cloud-based diagnostic platforms could further enhance scalability and accessibility, as evidenced by similar initiatives in other healthcare domains.

Further research should also explore multimodal fusion strategies, such as late fusion, attention-based fusion, or joint feature embedding, which may enable more effective integration of clinical and imaging representations. Such approaches could further enhance diagnostic precision and support more holistic Alzheimer’s disease assessment.

## 6 Conclusion

This study presents a dual-model diagnostic framework that combines an Artificial Neural Network (ANN) and a Convolutional Neural Network (CNN) to improve the detection and classification of Alzheimer’s disease. The ANN provides a rapid and accessible method for assessing patient risk using structured clinical data, while the CNN leverages MRI imaging to confirm the diagnosis and determine disease severity with high precision. Together, these models create a comprehensive diagnostic pathway that reflects real-world clinical workflows. The ANN achieved an accuracy of 87.08%, effectively identifying individuals at risk, whereas the CNN demonstrated 97% accuracy in staging Alzheimer’s disease. The incorporation of Grad-CAM visualizations further enhanced the interpretability of the CNN model, highlighting anatomically relevant regions and increasing clinician confidence in the system’s predictions.

The results underscore the potential of AI-driven multimodal approaches to strengthen early Alzheimer’s detection, support clinical decision-making, and facilitate timely intervention. Furthermore, repeated-run evaluations and confidence interval analyses support the reliability of the reported performance, emphasizing the robustness of the dual-model framework.

Future advancements could further expand the utility of this system. Integrating additional data modalities such as wearable sensor signals, longitudinal health data, or cognitive behavioral patterns may enhance early-stage detection. Exploring advanced multimodal fusion techniques, including attention-based and late-fusion strategies, could enable more effective integration of clinical and imaging representations. Optimizing CNN architectures for scalability, or deploying cloud-based inference pipelines, could also extend accessibility to resource-limited clinical environments. External validation with independent datasets such as ADNI or OASIS-3 represents an essential next step for strengthening generalizability and clinical applicability.

In summary, this dual-model system demonstrates the transformative potential of AI in Alzheimer’s diagnostics by providing an accurate, interpretable, and clinically meaningful framework for early disease detection and management.

## Data Availability

The clinical dataset used for the ANN model was obtained from the *“Alzheimer’s Disease Dataset (Classification)”* (39). The MRI dataset used for the CNN model was sourced from the *“Augmented Alzheimer MRI Dataset”* (40), which includes augmented MRI scans derived from the OASIS neuroimaging repository. Both datasets are publicly available and provided under open-access licenses. The implementation code is available from the authors upon reasonable request.
